# Transcriptional responses in the hepatopancreas of *Eriocheir sinensis* exposed to deltamethrin

**DOI:** 10.1371/journal.pone.0184581

**Published:** 2017-09-14

**Authors:** Zongying Yang, Yiliu Zhang, Yingying Jiang, Fengjiao Zhu, Liugen Zeng, Yulan Wang, Xiaoqing Lei, Yi Yao, Yujie Hou, Liangqing Xu, Chunxian Xiong, Xianle Yang, Kun Hu

**Affiliations:** 1 National Pathogen Collection Center for Aquatic Animals, Shanghai Ocean University, Shanghai, China; 2 Nanchang Academy of Agricultural Sciences, Nanchang, China; Zhejiang University College of Life Sciences, CHINA

## Abstract

Deltamethrin is an important pesticide widely used against ectoparasites. Deltamethrin contamination has resulted in a threat to the healthy breeding of the Chinese mitten crab, *Eriocheir sinensis*. In this study, we investigated transcriptional responses in the hepatopancreas of *E*. *sinensis* exposed to deltamethrin. We obtained 99,087,448, 89,086,478, and 100,117,958 raw sequence reads from control 1, control 2, and control 3 groups, and 92,094,972, 92,883,894, and 92,500,828 raw sequence reads from test 1, test 2, and test 3 groups, respectively. After filtering and quality checking of the raw sequence reads, our analysis yielded 79,228,354, 72,336,470, 81,859,826, 77,649,400, 77,194,276, and 75,697,016 clean reads with a mean length of 150 bp from the control and test groups. After deltamethrin treatment, a total of 160 and 167 genes were significantly upregulated and downregulated, respectively. Gene ontology terms “biological process,” “cellular component,” and “molecular function” were enriched with respect to cell killing, cellular process, other organism part, cell part, binding, and catalytic. Pathway analysis using the Kyoto Encyclopedia of Genes and Genomes showed that the metabolic pathways were significantly enriched. We found that the CYP450 enzyme system, carboxylesterase, glutathione-*S*-transferase, and material (including carbohydrate, lipid, protein, and other substances) metabolism played important roles in the metabolism of deltamethrin in the hepatopancreas of *E*. *sinensis*. This study revealed differentially expressed genes related to insecticide metabolism and detoxification in *E*. *sinensis* for the first time and will help in understanding the toxicity and molecular metabolic mechanisms of deltamethrin in *E*. *sinensis*.

## Introduction

Pyrethroids are synthetic pesticides, which were first derived from the extract of the flowers of *Chrysanthemum cinerariaefolium*, and they are widely used for agricultural and residential control of pest insects because of their selectivity and low toxicity against non-target organisms such as mammals and birds [1–4]. However, pyrethroids are highly toxic to fishes: LD_50_ for fishes is 10 to 1000 times less than the corresponding values for mammals and birds [5–8]; this means that pyrethroid contamination can make aquatic animals the targets of pyrethroid intoxication.

Pyrethroids have 2 subgroups because of their chemical structure: type I and II [9]. Deltamethrin,[(*S*)-cyano-(3-phenoxyphenyl)-methyl](1*R*,3*R*)-3-(2,2-dibromoethenyl)-2,2-dimethyl-cyclopropane-1-carboxylate, is a type II pyrethroid. Because of its short half-life in the environment and organisms, deltamethrin has become one of the most commonly used pesticides [10–12]. Many studies on the ecotoxicology of deltamethrin in fishes have been conducted [13–19], and the World Health Organization [20] determined that the lethal concentration of deltamethrin in fishes exposed for 96 h is 0.4–2 μg·L^-1^. Crustaceans are more sensitive to pyrethroids than fishes [21], and comparative tests have shown that deltamethrin is most toxic to crustaceans [22]. The 96-h LC_50_ value of deltamethrin for the pink shrimp (*Penaeus duorarum*) was 0.35 μg·L^-1^ [23], and Smith and Stratton [24] concluded that shrimps and lobsters are susceptible to all pyrethroids.

The Chinese mitten crab, *Eriocheir sinensis*, is an important crustacean species in China, and its culture in facilities was started in the early 1980s [25]. Because of an increasing demand for *E*. *sinensis* in the food market, commercial production is rapidly expanding, with an annual output worth approximately US$ 4 billion in Jiangsu Province, China [26]. The wide use of deltamethrin has posed a threat to crab breeding, as pyrethroids may be used for pond cleaning [27]. Biodegradation of pyrethroids in crustaceans has been reported [28]; however, there is currently no information on the effects of deltamethrin on *E*. *sinensis*, and the exact mode of action of deltamethrin in *E*. *sinensis* is unknown. Since alterations in gene expression after external stimuli are rapid, transcriptional responses to pharmaceutical drugs may help us to understand how an organism responds to a particular drug [29]. High-throughput RNA-sequencing (RNA-Seq) has become the conventional and highly effective technology for analyzing gene expression and identifying novel transcripts and differentially expressed genes [30]. RNA-Seq has been widely used to study various invertebrates, such as *E*. *sinensis* [31, 32], *Litopenaeus vannamei* [33], and *Crassostrea gigas* [34].

The hepatopancreas is not only a digestive gland but also an immune organ, and it plays an important role in the innate immune system and metabolism of xenobiotics. It is the primary site for synthesizing and excreting immune molecules, such as lectin or lectin-related proteins [35], antibacterial peptides [36] and beta-1,3-glucan-binding protein [37]. Transcriptome analysis of *L*. *vannamei* and *Litopenaeus setiferus* by expressed sequence tag analysis and novel gene discovery has revealed that the hepatopancreas plays a vital role in nonspecific immunity, and the cDNA library for the hepatopancreas is more representative than the library for hemocytes [35].

Li et al. [38] used RNA-Seq to construct a non-normalized cDNA library for the hepatopancreas in *E*. *sinensis* and identify immune-associated genes after bacterial infection. Huang et al. [39] assembled and annotated a comprehensive *de novo* transcriptome of *E*. *sinensis* hepatopancreas and characterized differentially expressed genes (DEGs) enriched at different molting stages. These studies were performed without a reference genome. However, Song et al. [40] performed, for the first time, genome sequencing, assembly, and annotation for *E*. *sinensis*. All the above-mentioned studies provide valuable information for studying important processes in *E*. *sinensis* or molecular mechanisms underlying the adaptive changes made by *E*. *sinensis* after stimulation with xenobiotics. The present study was conducted to analyze the transcriptome of the hepatopancreas in *E*. *sinensis* exposed to deltamethrin by using Illumina sequencing and bioinformatic analysis with the reference genome. The objective of this study was to annotate functional genes by using transcriptome analysis, analyze the short-term molecular effects of deltamethrin on *E*. *sinensis*, and evaluate the transcriptomic response of *E*. *sinensis* after exposure to deltamethrin. Our study helped in understanding the biological functions of the hepatopancreas of *E*. *sinensis* and provided a reference for further research on *E*. *sinensis*. Characterization of immune molecules and analysis of insecticide metabolism are crucial for healthy crab breeding and establishing guidelines for farmers to avoid inappropriate use of insecticides.

## Materials and methods

### Statement of ethics

This study was strictly performed in accordance with the guidelines on the care and use of animals for scientific purposes established by the Institutional Animal Care and Use Committee of Shanghai Ocean University, Shanghai, China.

### Maintenance and treatment of *E*. *sinensis*

In October 2016, healthy mature male crabs (average weight, 114.04 ± 9.14 g) were collected from the crab breeding base (32.864256110°N, 119.866714447°E) of Tao Huadao Agricultural Development Co., Ltd. in Anfeng Town, Xinghua City, northern Jiangsu Province, China. The crabs were transported to the laboratory in polystyrene boxes filled with cultivation water, which was aerated during transportation with an aeration pump. Upon arrival, the crabs were cultured in experimental tanks (capacity, 90 L) with 20 L of natural water under controlled temperature (23–25°C), pH (7.25 ± 0.25), and dissolved oxygen (6.85 ± 0.15 mg·L^-1^) for 2 weeks. During the acclimation period, the crabs were fed with a commercial crab feed (37% crude protein) 2 times a day and were starved 1 day before the experiment. After 2 weeks of acclimation, 60 healthy crabs were separated into 6 groups (3 test and 3 control groups); the crabs in the test groups were given a 40 min bath in 3 ppb of deltamethrin (95% purity, Sigma; dissolved in acetone to prepare a stock solution of 25 mg·L^-1^), and the crabs in the control groups were exposed to the same concentration of acetone as that of deltamethrin in the test groups. Each experiment was performed in triplicate. After 40 min, the crabs were dissected on ice, and the hepatopancreas was collected in liquid nitrogen and stored at -80°C for RNA extraction.

### RNA isolation, cDNA library construction, and sequencing

Total RNA was isolated from the hepatopancreas with TRIzol reagent (Invitrogen, USA), according to the manufacturer’s instructions. DNA contaminants were removed using RNase-free DNase I (TaKaRa Biotechnology, Dalian, China), and total RNA was eluted in 100 μL of RNase-free MilliQ H_2_O. Then, the RNA was stored at -80°C before the next process. The RNA quality was checked with the NanoDrop ND-1000 UV-vis Spectrophotometer (NanoDrop Technologies, Wilmington, DE, USA), and RNA integrity was determined using the RNA 6000 Nano LabChip kit (Agilent Technologies) and Agilent 2100 Bioanalyzer (Agilent Technologies, Palo Alto, CA, USA). We isolated poly(A) mRNA with oligo-dT beads and Oligotex mRNA kits (Qiagen). The mRNA was treated with fragmentation buffer, and the cleaved RNA fragments were used as templates to synthesize the first-strand cDNA with reverse transcriptase and random hexamer primers. The second-strand cDNA was synthesized with RNase H and DNA polymerase I. These double-stranded cDNA fragments were end-repaired with T4 DNA polymerase, Klenow fragment, and T4 polynucleotide kinase, followed by the addition of a single “A” base with Klenow 3′-5′ exopolymerase. Then, the fragments were ligated with an adapter or index adapter by using T4 quick DNA ligase. The adaptor-ligated fragments were selected on the basis of their size by using agarose gel electrophoresis. The desired cDNA fragment was excised from the gel, and PCR was performed to amplify the fragments. After validation using the Agilent 2100 Bioanalyzer and ABI StepOnePlus Real-Time PCR System (ABI, California, USA), the cDNA library was finally sequenced and constructed on a flow cell by using the high-throughput mode on the Illumina HiSeq 2500 unit (Illumina, San Diego, USA).

### Illumina sequencing, data processing, and quality control

The low-quality reads were filtered out and 3′ adapter sequences were removed using Trim Galore. We cleaned the obtained reads by using FastQC software (http://www.bioinformatics.babraham.ac.uk/projects/fastqc/), and content and quality of the remaining clean reads were evaluated. Then, we performed a comparative analysis with the reference genome of *E*. *sinensis*. For each sample belonging to the test and control groups, sequence alignment with the reference genome sequences was conducted using TopHat [41].

### Identification of DEGs

To evaluate the expression levels of the transcripts in different groups, we used RSEM software with default parameter settings [42] to estimate the expression level (relative abundance) of a specific transcript with fragments per kilobase of transcript per million fragments mapped (FRKM) [43]. The expression level of each transcript was transformed using base log_2_ (FPKM+1). We used DESeq software to screen the DEGs and calculate the fold change of the transcript [44]. Two-fold changes were used to investigate the expression, and *p*-values < 0.05 were considered statistically significant.

### GO functional annotation and enrichment analysis for DEGs

To analyze the potential functions of the DEGs, we first annotated the DEGs against the UniProt database (http://www.uniprot.org/). Then, we analyzed the functional annotation by using gene ontology terms (GO; http://www.geneontology.org) with Blast2GO (https://www.blast2go.com/) [45]. All the DEGs were mapped to GO terms in the GO database, and the number of genes for every term was calculated. The ultra-geometric test was used to detect the enriched GO terms of DEGs when compared with the transcriptome background. The formula used was as follows:

$$P=1-\sum_{i=0}^{m-1}\frac{\left(\begin{array}{l} M\\ i \end{array}\right)\left(\begin{array}{l} N-M\\ n-i \end{array}\right)}{\left(\begin{array}{l} N\\ n \end{array}\right)}$$

where, *N* represents the number of genes with GO annotation added; *n* is the number of DEGs in *N*; *M* is the number of genes annotated to specific GO terms; and *m* is the number of DEGs in *M*. The calculated *p-*value was subjected to Bonferroni correction. A corrected *p-*value of 0.05 was determined as the threshold of statistical significance, and GO terms were considered significantly enriched in the DEGs when the corrected *p-*value was <0.05.

### Pathway analysis of DEGs

Pathways of the DEGs were annotated using blastall (http://nebc.nox.ac.uk/bioinformatics/docs/blastall.html) against the Kyoto Encyclopedia of Genes and Genomes (KEGG) database. We identified the enriched DEG pathways with the same formula as that used for the GO analysis. *N* represents the number of genes added with KEGG annotation; *n* represents the number of DEGs in *N*; *M* represents the number of genes annotated to specific pathways; and *m* is the number of DEGs in *M*.

### Quantitative reverse-transcription PCR verification

We used quantitative reverse-transcription (qRT)-PCR to verify the expression levels of DEGs identified using RNA-Seq analysis. The primers were designed with Primer 5 software, and β-actin of *E*. *sinensis* was used as the internal control to normalize the expression levels. All the experiments were performed in triplicate. The reaction was performed using a 25 μL volume comprising 2 μL of cDNA, 12.5 μL of SYBR Premix Ex Taq (TaKaRa), 9.5 μL of RNase-free H_2_O, and 0.5 μL of the forward and reverse primers (10 μmol·L^-1^). The thermal cycling program was as follows: 95°C for 30 s, followed by 40 cycles of 95°C for 5 s, 60°C for 30 s, and 72°C for 30 s. The melting curve analysis was conducted at the end of qRT-PCR to confirm PCR specificity. The expression levels were analyzed with the 2^—ΔΔ^ CT method.

## Results

### Illumina sequencing and quality assessment

RNA-Seq was conducted using Illumina sequencing to examine the effects of deltamethrin on *E*. *sinensis* transcriptome. The raw sequence reads in the test samples were 92,094,972, 92,883,894, and 92,500,828, and those in the control samples were 99,087,448, 89,086,478, and 100,117,958. After filtering and checking the quality, the trimmed reads in the test samples were 77,649,400, 77,194,276, and 75,697,016, and those in the control samples were 79,228,354, 72,336,470, and 81,859,826. The trim rates of the test samples were 84.31%, 83.11%, and 81.83%, and those of the control samples were 79.96%, 81.20%, and 81.76%; the mean length was 150 bp (Table 1). The results showed that the transcriptome sequencing of *E*. *sinensis* was successful, and the trimmed reads could be used for further analyses.

**Table 1:** Summary of reads obtained from *E*. *sinensis* transcriptome sequencing.

Sample	Raw reads	Trimmed reads	Rate of clean Q30 bases (%)	Average length (bp)	Trim rate (%)
Control 1	99,087,448	79,228,354	94.01	150	79.96
Control 2	89,086,478	72,336,470	94.32	150	81.20
Control 3	100,117,958	81,859,826	94.31	150	81.76
Test 1	92,094,972	77,649,400	94.77	150	84.31
Test 2	92,883,894	77,194,276	94.58	150	83.11
Test 3	92,500,828	75,697,016	94.42	150	81.83

### Comparative analysis with the reference genome

The trimmed reads from the hepatopancreas transcriptome of *E*. *sinensis* were compared with the reference genome sequence. The total mapped rates of the reads were 63.00%, 64.00%, and 63.00% in the 3 test groups and 63.00%, 64.00%, and 60.00% in the 3 control groups. Multiple mapped reads were about 3 million (3,030,201), 2 million (2,430,849), and 3 million (3,366,219) for the 3 test groups, and 3 million (3,791,979), 2 million (2,430,849), and 3 million (3,201,152) for the 3 control groups, accounting for 4.00%, 3.00%, 4.00%, 5.00%, 5.00%, and 4.00% of the total reads. There were 28,972,914, 28,138,541, and 28,200,925 unmapped reads in the test groups, and 29,285,518, 26,016,203, and 32,444,243 unmapped reads in the control groups (Table 2).

**Table 2:** Statistical results for mapping the trimmed reads with the reference genome.

Map to genome		Total reads	Mapped reads	Unmapped reads	Multiple mapped reads
Control 1	Read numbers	79,228,354	49,942,836	29,285,518	3,791,979
	Percentage	100.00%	63.00%	36.96%	5.00%
Control 2	Read numbers	72,336,470	46,320,267	26,016,203	3,627,885
	Percentage	100.00%	64.00%	35.97%	5.00%
Control 3	Read numbers	81,859,826	49,415,583	32,444,243	3,201,152
	Percentage	100.00%	60.00%	39.63%	4.00%
Test 1	Read numbers	77,649,400	48,676,486	28,972,914	3,030,201
	Percentage	100.00%	63.00%	37.31%	4.00%
Test 2	Read numbers	77,194,276	49,055,735	28,138,541	2,430,849
	Percentage	100.00%	64.00%	36.45%	3.00%
Test 3	Read numbers	75,697,016	47,496,091	28,200,925	3,366,219
	Percentage	100.00%	63.00%	37.26%	4.00%

### Analysis of DEGs

**Fig 1:**
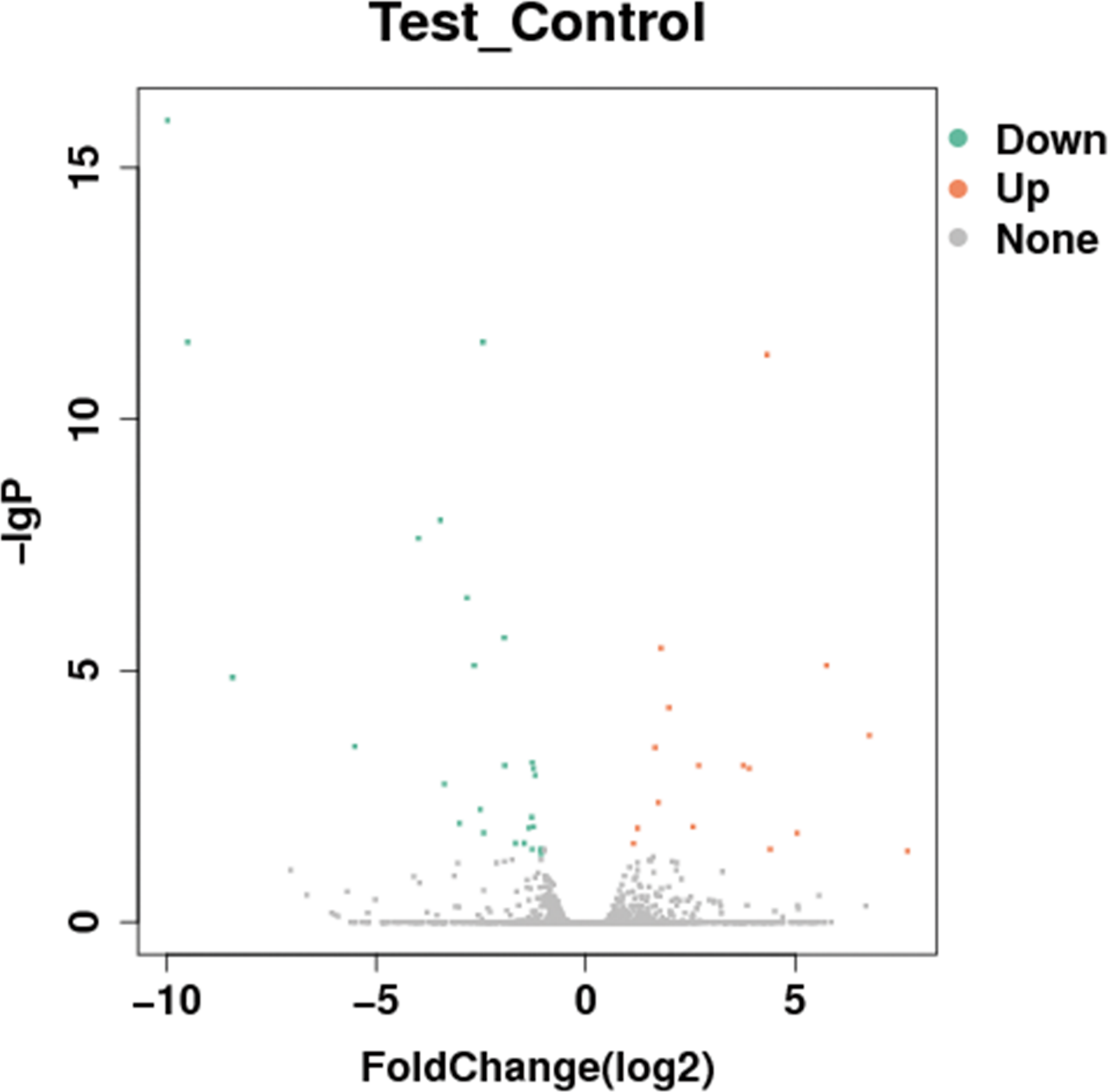
Effects of deltamethrin treatment on the gene expression profile of *E*. *sinensis*. Volcanic plot of the degree of differences in the expression profile of *E*. *sinensis*. X-axis, log_2_ (fold change); Y-axis, -log_2_ (p-value). Red, significantly upregulated genes; green, significantly downregulated genes. Each dot represents one gene.

**Fig 2:**
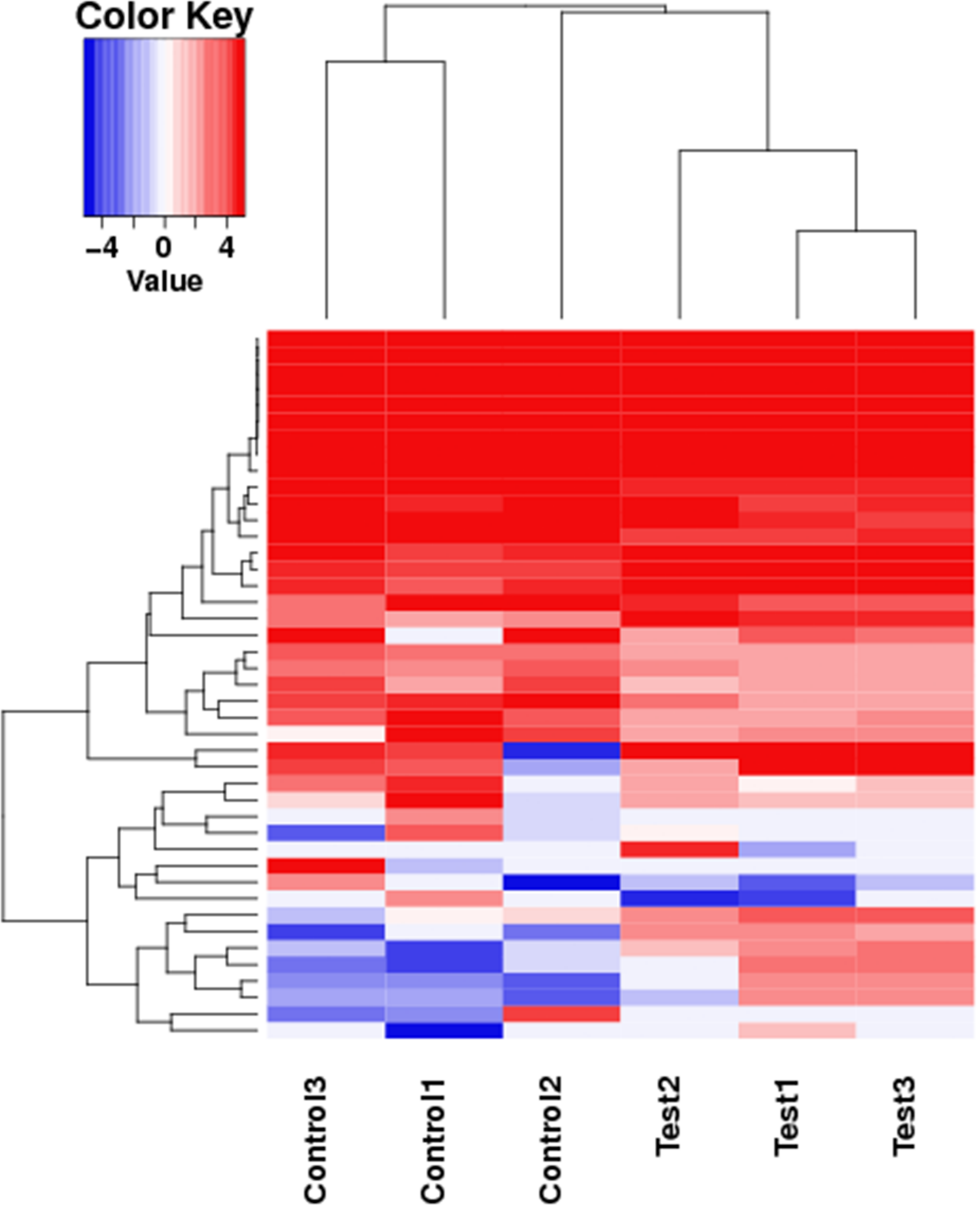
Effects of deltamethrin treatment on the gene expression profile using pattern clustering. Red, upregulated genes; green, downregulated genes. Each line represents one gene.

To investigate the DEGs of *E*. *sinensis* exposed to deltamethrin, the Cuffdiff program was used to generate the gene expression profiles (Figs 1 and 2). This program identified 160 significantly upregulated DEGs and 167 significantly downregulated DEGs. The results indicated that deltamethrin affected gene expression in *E*. *sinensis*.

### GO annotation of DEGs

**Fig 3:**
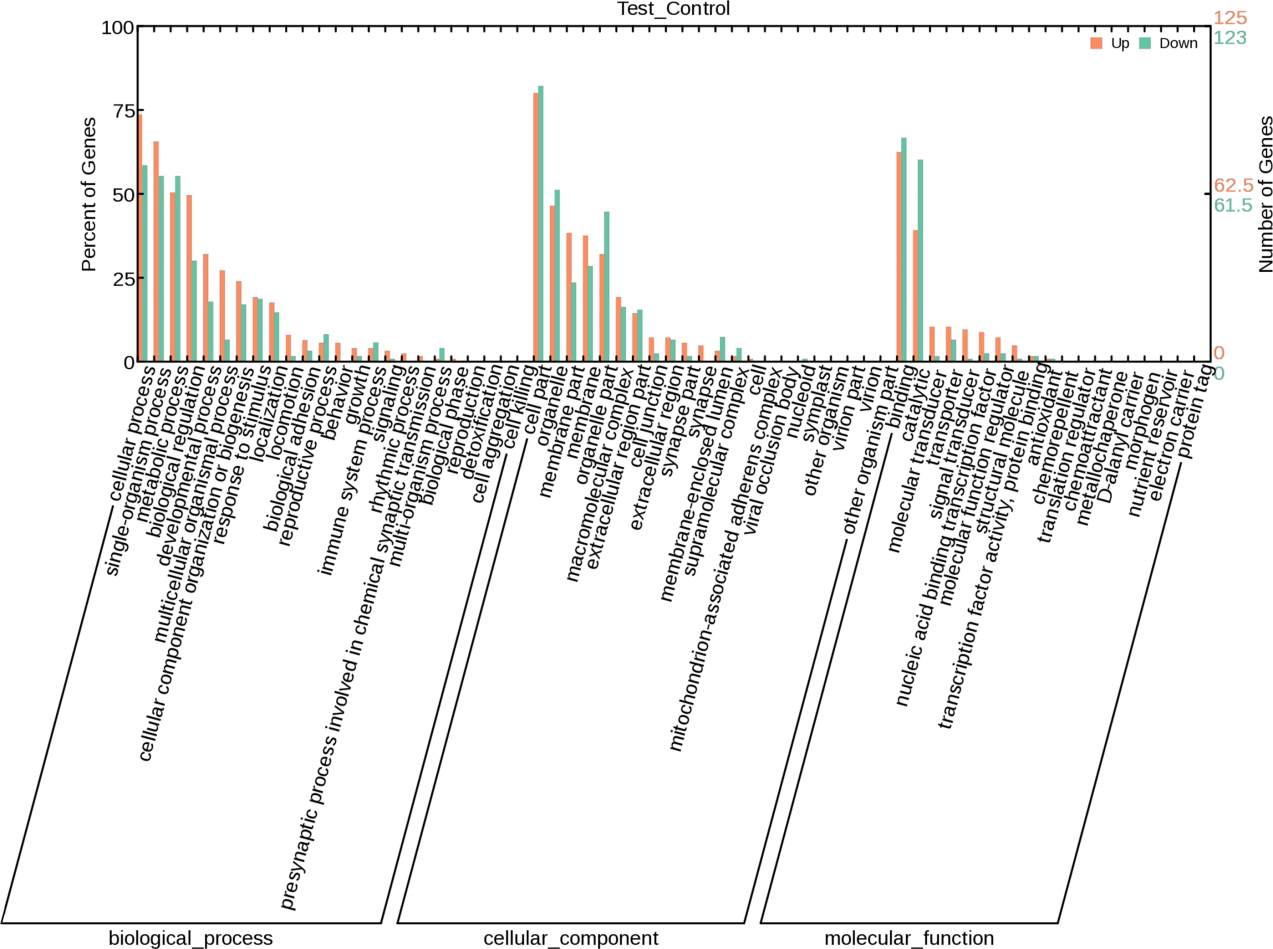
Histogram of the enriched subcategories after gene ontology (GO) annotation of the differentially expressed genes (DEGs) in *E*. *sinensis* specimens that received deltamethrin treatment. The GO terms (X-axis) were grouped into 3 main ontologies: biological process (BP), cellular component (CC), and molecular function (MF). The Y-axis indicates the number of DEGs.

We classified 327 non-redundant unigenes into 65 GO terms under 3 domains: biological process, cellular component, and molecular function. The dominant subcategories of biological process were “cellular process” (164 genes), “single-organism process” (150 genes), “metabolic process” (131 genes), and “biological regulation” (99 genes). The dominant subcategories of cellular component were “cell part” (201 genes), “organelle” (121 genes), “organelle part” (95 genes), and “membrane” (82 genes). The dominant subcategories of molecular function were “binding” (160 genes), “catalytic” (123 genes), “transporter” (21 genes), and “molecular transducer” (15 genes) (Fig 3).

### KEGG pathway analysis of DEGs

**Fig 4:**
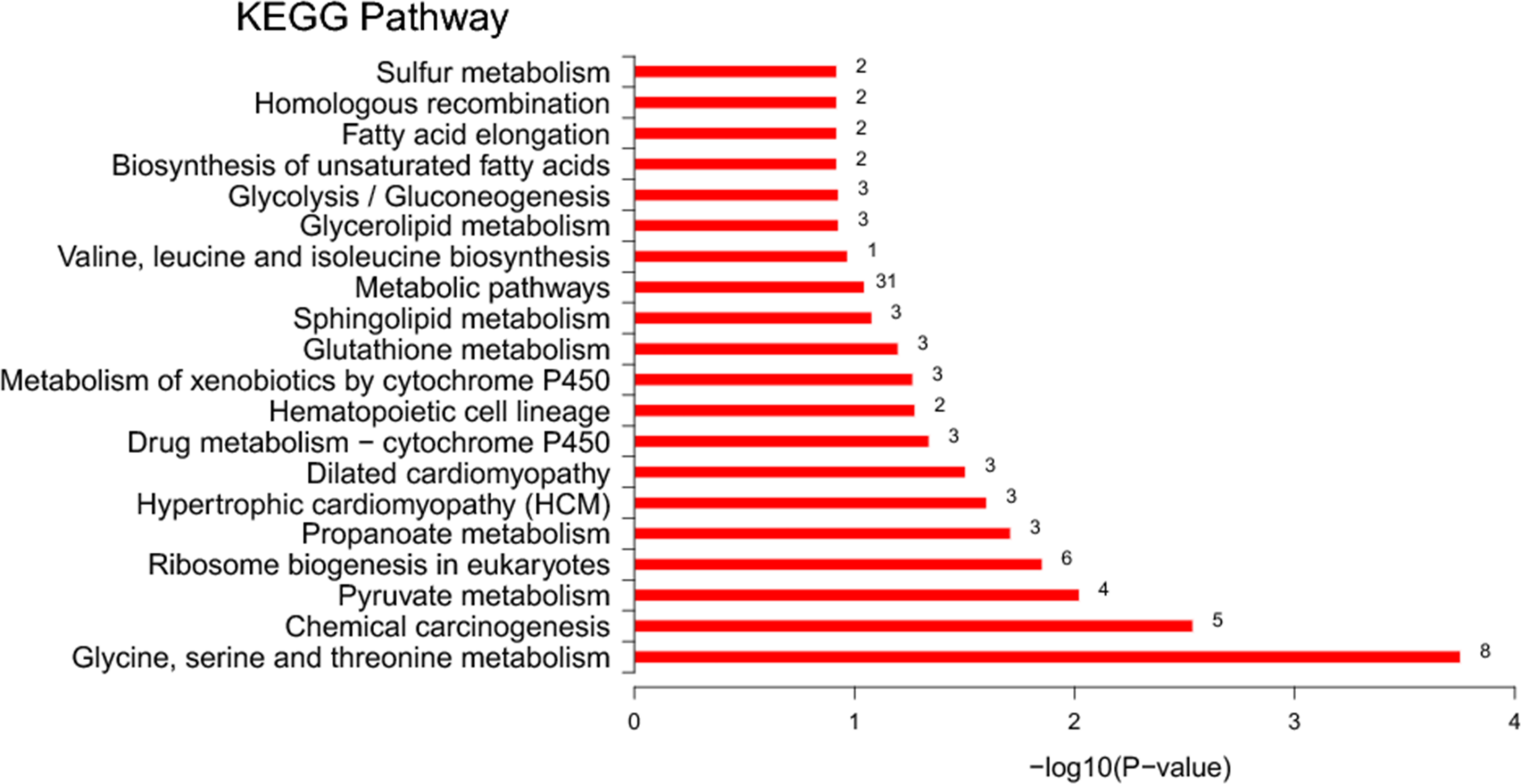
Histogram of the top 20 most-enriched KEGG pathways of DEGs in *E*. *sinensis* after deltamethrin treatment. Y-axis, KEGG pathway categories; X-axis, statistical significance of the enrichment.

The DEGs were mapped to the KEGG database to further investigate the biological functions and important pathways on the basis of the whole transcriptome background; 327 unigenes were mapped to 262 pathways. Some genes were found in multiple pathways, and some genes were restricted to a single pathway. The first 20 most-enriched KEGG pathways of DEGs were noted; metabolic pathways (31 genes) were the most significantly enriched pathways, followed by glycine, serine, and threonine metabolism (8 genes); ribosome biogenesis in eukaryotes (6 genes); chemical carcinogenesis (5 genes); pyruvate metabolism (4 genes); drug metabolism-cytochrome P450 (3 genes); metabolism of xenobiotics by cytochrome P450 (3 genes); and glycerolipid metabolism (3 genes) (Fig 4).

### DEGs involved in metabolic pathways

Metabolic pathways, biosynthesis of amino acids, and biosynthesis of secondary metabolites are all metabolism-related biological pathways. In our study, the lipid metabolism-related pathways included glycerophospholipid metabolism (1 DEG), glycerolipid metabolism (3 DEGs), steroid biosynthesis (2 DEGs), fatty acid biosynthesis (1 DEG), fatty acid elongation (2 DEGs), biosynthesis of unsaturated fatty acids (2 DEGs), synthesis and degradation of ketone bodies (1 DEG), fatty acid degradation (2 DEGs), fat digestion and absorption (1 DEG), and steroid hormone biosynthesis (2 DEGs). The DEGs involved in the lipid metabolism-related pathways were downregulated, except for VN_GLEAN_10002420|K11262 (fatty acid biosynthesis) and VN_GLEAN_10004144|K13368 (steroid hormone biosynthesis). The DEGs involved in glutathione metabolism (3 DEGs) were all downregulated. Besides the effect of deltamethrin on lipid and glutathione metabolism, we found that deltamethrin treatment affected carbohydrate metabolism, including glycolysis/gluconeogenesis (3 DEGs), pentose phosphate pathway (1 DEG), amino sugar and nucleotide sugar metabolism (3 DEGs), and fructose and mannose metabolism (2 DEGs). We should point out that the DEGs of the pentose phosphate pathway were upregulated, which may indicate that the metabolism of deltamethrin is an energy consumption process. We also found that deltamethrin had an effect on protein metabolism: the DEGs involved in ubiquitin-mediated proteolysis (2 DEGs) were downregulated, while the DEGs involved in protein digestion and absorption (2 DEGs) were upregulated.

The drug metabolic pathways were composed of metabolism of xenobiotics by cytochrome P450 (3 DEGs) and drug metabolism-other enzymes (2 DEGs). Interestingly, all the DEGs were downregulated. These findings indicated that deltamethrin treatment not only affected material (carbohydrate, lipid, protein, and other substances) metabolism but also detoxification mechanism of the hepatopancreas.

### DEGs involved in signal transduction

We found that many DEGs were associated with signal transduction pathways. The DEGs involved in the calcium signaling pathway (1 DEG), axon guidance (3 DEGs), MAPK signaling pathway (2 DEGs), ABC transporters (1 DEG), glutamatergic synapse (2 DEGs), and GABAergic synapse (2 DEGs) were all upregulated.

Deltamethrin is neurotoxic, and it affects the nervous system by modifying the Na^+^ channels to remain open, resulting in prolonged Na^+^ transport along the membranes of nerve cells. The DEGs of axon guidance were all upregulated and may be associated with the toxic mechanism of deltamethrin.

The MAPK signaling pathway can be found in all eukaryotes, and mitogen-activated protein kinase signaling cascades include extracellular signal-regulated kinase, MAPK kinase, and MAPK kinase kinase. Most of the information on the MAPK signaling pathway is focused on vertebrates. In vertebrates, this pathway is significant for anti-stress, inflammation, cell development, reproduction, and differentiation. The MAPK signaling pathway transducts the extracellular signal to the cytoplasm and nucleus by substrate phosphorylation, and the physiological process is controlled by a conserved kinase cascade [46]. However, there is limited information on the MAPK signaling pathway in aquatic invertebrates. To date, Lin et al. [47] have found that the MAPK signaling pathway influenced *Trichomonas vaginalis*-induced proinflammatory cytokines in the shrimp *Penaeus monodon*; Feld et al. [48] found that the MAPK signaling pathway participated in neural plasticity in the crab *Chasmagnathus*; Zhu et al. [49] reported that the MAPK signaling pathway was involved in enrofloxacin metabolism in *E*. *sinensis*; and Li et al. [38] found that the MAPK signaling pathway played an important role in microbe-challenged *E*. *sinensis*. In our study, we found that the MAPK signaling pathway was associated with deltamethrin metabolism, and the expression levels of the genes involved in the pathway were upregulated. The results showed that the MAPK signaling pathway is involved in xenobiotic metabolism; however, the mechanism of action needs to be studied further.

### Differential expression verification of DEGs

**Fig 5:**
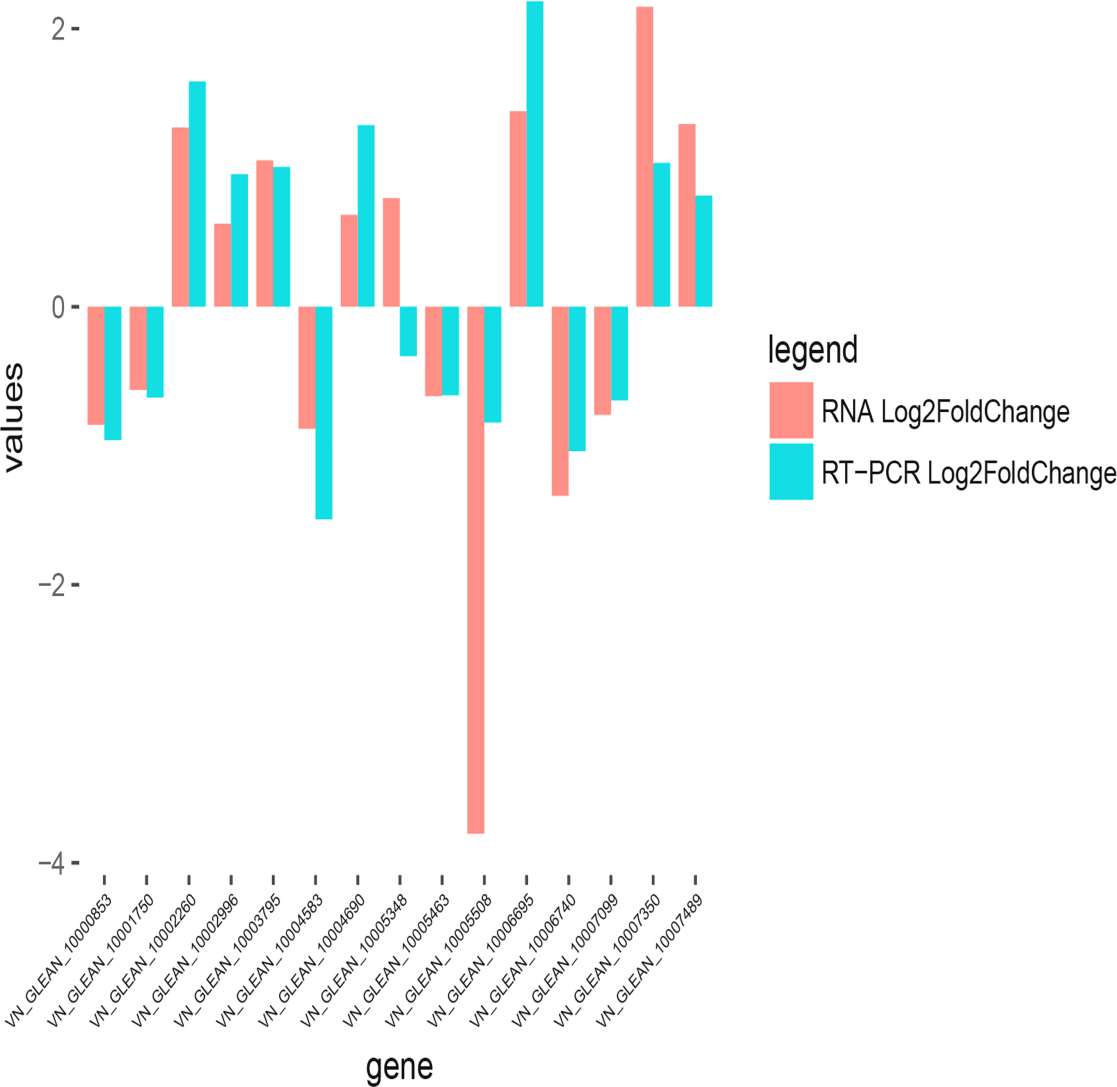
Comparison of 15 gene expression levels by using RNA-Seq and RT-PCR. Negative values indicate that the gene expression in *E*. *sinensis* was downregulated after deltamethrin treatment; positive values indicate that the gene expression was upregulated.

qRT-PCR was used to verify the gene expression profiles, and 15 genes were suggested to be related to the deltamethrin treatment after GO and KEGG analyses. The primer sequences for all the examined genes are listed in Table 3. The qRT-PCR verification results for the 15 genes were consistent with the results of RNA-Seq, except for 1 gene (VN_GLEAN_10005348; Fig 5). These results indicate that the RNA-Seq results are generally reliable, and further experiments are required to verify the results of this study.

**Table 3:** Oligonucleotide primers of qRT-PCR for validation of DEGs.

Gene name	Predict function	Go category	Pathway name	Nucleotide sequence (5′-3′)	Expected product
ACTIN	—	—	—	F-TCACACACTGTCCCCATCTAG	114bp
				R-ACCACGCTCGGTCAGGATTTTC	
VN_GLEAN_10007350	Thrombospondin-3	Calcium ion binding (Molecular function)	Phagosome	F-CACGGGCGATACTGAGAACC	129bp
				R-ATACGGAGGCGAATGAGACC	
VN_GLEAN_10000853	Long-chain-fatty-acid-CoA ligase 4	Cytoplasm (Cellular component)	Peroxisome	F-GGACCTCGTGAAGTTGAAGCA	100bp
				R-GGCGAAGACGCAGATGTTG	
VN_GLEAN_10007099	1-acyl-sn-glycerol-3-phosphate acyltransferase delta	|1-acylglycerol-3-phosphate O-acyltransferase activity (Molecular function)	Glycerophospholipid metabolism	F-GGTGGTCTGGCTCTGATGTG	136bp
				R-CTCCGATCAGCGACAATCC	
VN_GLEAN_10005463	Glutathione synthetase	glutathione synthase activity (Molecular function)	Glutathione metabolism	F-GATGTGATGCTGACCAAGTGC	107bp
				R-CCAAATCCTGAGGCGATAGTG	
VN_GLEAN_10001750	Glutathione S-transferase theta-1	Glutathione transferase activity (Molecular function)	Glutathione metabolism	F-ATCTTATGTCTCAGCCCTCACG	119bp
				R-TGTTGCATATTCCTTGGTGTAGTG	
VN_GLEAN_10005348	Mitogen-activated protein kinase kinase kinase 4	MAP kinase kinase kinase activity (Molecular function)	MAPK signaling pathway	F-GGCTGGCATTGTAGGGACC	175bp
				R-GCCCACTCGGAACATTATCTG	
VN_GLEAN_10004690	ATP-binding cassette sub-family A member 3	Cellular protein metabolic process (Biological process)	ABC transporters	F-GCCTGAGCATAGAGGGAAACC	100bp
				R-TTGTCAGCAACAGTCAGAGCC	
VN_GLEAN_10006695	Voltage-dependent L-type calcium channel subunit alpha-1C	Caveolar macromolecular signaling complex (Cellular component)	Calcium signaling pathway	F-GGTCACTCTTCTCAGGAAAATCTC	129bp
				R-GCATTGAACATTACTGTCCCATC	
VN_GLEAN_10004583	Cytochrome P450 3A11	Oxidoreductase activity (Molecular function)	Drug metabolism-other enzymes	F-AGTCAGCGTTGGCTCTATGTG	120bp
				R-GTGTTTGAGGAGGTCAGGGTC	
VN_GLEAN_10006740	Carboxylesterase 1C	Carboxylic ester hydrolase activity (Molecular function)	Drug metabolism-other enzymes	F-CGACGCAAGAAGAGTGACGA	158bp
				R-TAAGCAGGCTGAGGGGACC	
VN_GLEAN_10005508	Probable glutathione S-transferase 5	|Glutathione transferase activity (Molecular function)	Drug metabolism-cytochrome P450	F-GGCAACATCCCCTTCACG	171bp
				R-CCATTCATTCCCCTCAAACC	
VN_GLEAN_10002260	Homer protein homolog 2	|G-protein coupled glutamate receptor binding (Molecular function)	Glutamatergic synapse	F-GCTCTTGCTCAAAGTTCTGCC	159bp
				R-GCGACTGTTCTCCTCCTTGTAG	
VN_GLEAN_10007489	Sodium-and chloride-dependent GABA transporter 1	gamma-aminobutyric acid (Molecular function)	GABAergic synapse	F-CAGATTTCCGACGGCATTG	113bp
				R-GTCCACTTCACTCCCTTCCAG	
VN_GLEAN_10002996	Fructose-bisphosphate aldolase	Fructose-bisphosphate aldolase activity (Molecular function)	Pentose phosphate pathway	F-GAACACCCCAAGCTACCAGG	120bp
				R-TCGCCGTCAGGAAGAACC	
VN_GLEAN_10003795	b(0,+)-type amino acid transporter 1	Amino acid transport (Biological process)	Protein digestion and absorption	F-CACTCATTATGGTGATCCCGTC	123bp
				R-CGTCAGGCATGGTGTAGCG	

## Discussion

Deltamethrin (3 ppb) is often applied using a 30–40 min bath [10, 50]. We designed this study to evaluate the effects of deltamethrin on *E*. *sinensis*, which is a very economically important crab species in China. Transcriptome sequencing has become a powerful technique for studying the mechanisms underlying changes in the biological characteristics of an organism. When compared with the traditional methods of analyzing the hepatopancreas of *E*. *sinensis*, our study produced more sequencing reads [51, 52] and was useful in yielding genomic resources and molecular information on *E*. *sinensis* exposed to deltamethrin.

### Detoxification mechanism of the hepatopancreas

Deltamethrin is a type II pyrethroid used against ectoparasites. Biodegradation of pyrethroids is principally catalyzed by P450 enzymes, carboxylesterases, and glutathione-*S*-transferase (GST) [10]. Biodegradation of deltamethrin occurs through hydrolysis of the central ester via carboxylesterases [9] and oxidation by cytochrome P450 enzymes [53]; however, the metabolites are different in different species [54]. Wheelock et al. [55] found that carboxylesterase activity was important for removing pyrethroid toxicity and could be used as an indicator for pyrethroids during toxicity identification.

Biodegradation of pyrethroids through oxidation by increasing the levels of reactive oxygen species (ROS) has been reported in crustaceans and fishes [15, 28]. Hence, elimination of ROS can alleviate oxidative stress and subsequently decrease toxicological damage [56]. Transcriptomic expression of the genes related to the antioxidant system, including non-enzymatic and enzymatic antioxidants, plays an important role in protecting organisms from damage. It is important to note that GST belongs to the group of transferases responsible for the biodegradation of pesticides, and the activity of this enzyme is associated with the metabolism of insecticides [57]. These transferases catalyze the conjugation of electrophilic compounds to the thiol group of reduced glutathione, producing more soluble products for excretion and, hence, directly decreasing the levels of this antioxidant [58]. We found that the expression levels of CYP450 and glutathione metabolism genes were downregulated, which means that CYP450 and glutamathione are relevant to deltamethrin metabolism. Our results were consistent with those of previous studies: Banka et al. [59] found that CYP450 enzymes were inhibited by deltamethrin in vivo and in vitro, and Abdel-Daim et al. [60] revealed that deltamethrin could induce lipid peroxidation and glutathione reduction. However, Erdogan et al. [61] found a significant increase in CYP450 in the muscle of rainbow trout exposed to deltamethrin, and Olsvik et al. [62] reported that the levels of non-enzymatic antioxidant glutathione were increased in all the tissues exposed to deltamethrin. The reasons for these discrepancies are unclear, but differences in exposure duration, deltamethrin concentration, and different species and tissues may explain the findings.

### Candidate genes involved in energy and material metabolism

In our study, we found that the gene involved in the pentose phosphate pathway was upregulated, which indicated that deltamethrin disrupted carbohydrate metabolism. Abdel-Daim et al. [60] showed that serum glucose and cholesterol levels were significantly increased because of deltamethrin toxicity in the Nile tilapia, and El-Sayed & Saad [63] found that a reduction in hepatic glycogen levels accompanied by an increase in plasma glucose levels was a common reaction against toxic insult followed by metabolic stress in fishes. The hyperglycemic effect after pyrethroid treatment in the Nile tilapia suggests the effects of deltamethrin on the glycolytic and glycogenesis pathways [64]. Our study also confirmed that deltamethrin interfered with carbohydrate metabolism. Lipids are stored in droplets and consist of a triglyceride core surrounded by a layer of phospholipids and embedded proteins, and they represent a major component of the fat body and main source of metabolic fuel [65]. Phospholipids are a major component of biomembranes, and most pesticide preparations are characterized by high lipophilicity. The cell membrane is the principal site where pesticides act, and Cengiz et al. [66] found that deltamethrin increased the peroxidation of structurally important unsaturated fatty acids within the phospholipid structure of the membrane, resulting in cell membrane damage. In the enrichment analysis in our study, “Glycerophospholipid metabolism” and “Glycerolipid metabolism” were the important pathways related to lipid metabolism in *E*. *sinensis*, and the genes involved in the 2 pathways and biosynthesis of unsaturated fatty acids were all downregulated. We found that deltamethrin not only disrupted lipid storage and mobilization but also influenced protein metabolism. The genes involved in protein digestion and absorption were upregulated, and the genes involved in ubiquitin-mediated proteolysis were downregulated. Thus, deltamethrin disrupted carbohydrate, lipid, and protein metabolism, which would induce abnormalities in metabolism and nutrient absorption.

### Effects on signal transduction in deltamethrin

The main mechanism of deltamethrin as a pesticide is believed to result from its binding to a specific receptor site on voltage-dependent Na^+^ channels, leading to prolonged Na^+^ transport along the membranes of nerve cells and neural hyperexcitation [5]. In this study, we found that the genes of the Na^+^- and Cl^—^dependent GABA transporter were upregulated. Besides, the genes involved in glutamatergic synapse, calcium signaling pathway, and cholinergic synapse were all upregulated.

Previous studies have revealed that many target sites other than the Na^+^ channel may be linked to the toxicity of deltamethrin. The voltage-dependent Cl^-^ channel has been proposed as a target of pyrethroids [67]. Voltage-sensitive Cl^-^ channels are found in many tissues, and their function is to control membrane excitability. Na^+^ and Cl^-^ conductance has reciprocal effects on membrane excitability [68]. A previous study has shown that pyrethroids can act on GABA-gated Cl^-^ channels [69], and this effect probably contributes to the seizures that accompany severe type II poisoning. Several other studies have suggested the role of the GABA_A_ receptor–ionophore complex in type II pyrethroid toxicity [70, 71]. Glutamate is an important excitatory neurotransmitter; l-glutamate activates several subtypes of receptors, leading to an increase in intracellular calcium-ion concentration. Many responses to glutamate in the central nervous system can be directly attributed to these changes in membrane polarization and calcium ions. Radcliffe & Dani [72] reported that strong, brief stimulation of nicotinic acetylcholine receptors enhanced hippocampal glutamatergic synaptic transmission on 2 independent time scales and altered the relationship between consecutively evoked synaptic currents. In this study, we also found that deltamethrin could increase glutamatergic synaptic transmission. Acetylcholinesterase activity (AChE) has also been used as a biomarker for pyrethroids [73]; AChE plays a dominant role in cholinergic neurotransmission, hydrolyzing the neurotransmitter acetylcholine at the cholinergic synapses [74]. Tu et al. [28] reported inhibition of AChE in the black tiger shrimp exposed to deltamethrin; we also found that the genes involved in cholinergic synapse were upregulated. Above all, deltamethrin affected the nerve impulse transmissions.

Pyrethroids primarily act on the central nervous system. Researchers may be confused about their exact mechanism of action because pyrethroids interact with not only receptors but also a wide range of ion channels. Nevertheless, it is clear that pyrethroids are likely to interact reversibly with ion channels by their phosphorylation state, and the Na^+^ channels are a major target.

## Conclusions

Because of the rapid metabolism and low toxicity of deltamethrin in non-target animals and humans, it has been widely used in many countries against pests [18, 56]. However, because of its properties, deltamethrin can cause enormous damage to aquatic animals. Alterations in the expression levels of CYP450, carboxylesterase, and GST could disturb the metabolization of deltamethrin, which may cause imbalances in detoxification processes and modifications in the synthesis and degradation of endogenous molecules with key biological activities.

In the present study, deltamethrin suppressed the gene expression levels of the antioxidant enzymes and glutathione. Deltamethrin is neurotoxic, and it also disrupted the metabolism of carbohydrates, lipids, and proteins in *E*. *sinensis*, affecting nutrient absorption and metabolization and making *E*. *sinensis* vulnerable to pathogen infections and environmental changes. In this study, we have elucidated the genes that were regulated when *E*. *sinensis* was exposed to deltamethrin; however, further studies are required to reveal the specific mechanism underlying deltamethrin detoxification, especially with respect to post-transcriptional processes.

### Data archiving

The sequencing reads are available in the NCBI SRA database (SRP105235).

## Supporting information

S1 Table Differentially expressed genes. (XLS)

S2 Table GO analysis of the differentially expressed genes. (XLS)

S3 Table KEGG pathways of all unigenes. (XLS)

S4 Table Top 20 most-enriched KEGG pathways of the differentially expressed genes. (XLS)

S5 Table Validation results of 15 differentially expressed genes by RT-PCR. (XLS)
